# Functional diversification of *Flowering Locus T* homologs in soybean: *GmFT1a* and *GmFT2a/5a* have opposite roles in controlling flowering and maturation

**DOI:** 10.1111/nph.14884

**Published:** 2017-11-09

**Authors:** Wei Liu, Bingjun Jiang, Liming Ma, Shouwei Zhang, Hong Zhai, Xin Xu, Wensheng Hou, Zhengjun Xia, Cunxiang Wu, Shi Sun, Tingting Wu, Li Chen, Tianfu Han

**Affiliations:** ^1^ MOA Key Laboratory of Soybean Biology (Beijing) Institute of Crop Science The Chinese Academy of Agricultural Sciences 12 Zhongguancun South Street Beijing 100081 China; ^2^ Key Laboratory of Soybean Molecular Design Breeding Northeast Institute of Geography and Agroecology Chinese Academy of Sciences Harbin 150081 China

**Keywords:** flowering inhibitor, Flowering Locus T (FT) homolog, *GmFT1a*, maturation, soybean

## Abstract

- Soybean flowering and maturation are strictly regulated by photoperiod. Photoperiod‐sensitive soybean varieties can undergo flowering reversion when switched from short‐day (SD) to long‐day (LD) conditions, suggesting the presence of a ‘floral‐inhibitor’ under LD conditions.
- We combined gene expression profiling with a study of transgenic plants and confirmed that *GmFT1a*, soybean *Flowering Locus T* (*FT*) homolog, is a floral inhibitor.
- *GmFT1a* is expressed specifically in leaves, similar to the flowering‐promoting *FT* homologs *GmFT2a/5a*. However, in Zigongdongdou (ZGDD), a model variety for studying flowering reversion, *GmFT1a* expression was induced by LD but inhibited by SD conditions. This was unexpected, as it is the complete opposite of the expression of flowering promoters *GmFT2a/5a*. Moreover, the key soybean maturity gene *E1* may up‐regulate *GmFT1a* expression. It is also notable that *GmFT1a* expression was conspicuously high in late‐flowering varieties. Transgenic overexpression of *GmFT1a* delayed flowering and maturation in soybean, confirming that *GmFT1a* functions as a flowering inhibitor.
- This discovery highlights the complex impacts of the functional diversification of the *FT* gene family in soybean, and implies that antagonism between flowering‐inhibiting and flowering‐promoting *FT* homologs in this highly photoperiod‐sensitive plant may specify vegetative vs reproductive development.

## Introduction

Multiple exogenous signals regulate whether plants undertake vegetative or reproductive growth. Such signals include changes in day length (photoperiod). So‐called ‘inductive’ photoperiodic conditions that are perceived in the leaves can cause plants to transition from vegetative to reproduce growth by activating the expression of flowering time genes, especially the homologs of the *Flowering Locus T* (*FT*) gene. It is accepted that FT is at least part of the long‐sought ‘florigen’ signal (Zeevaart, 2006). The FT protein interacts with the bZIP transcription factor Flowering Locus D (FD) in the shoot apical meristem to regulate the initiation of flowering (Abe *et al*., 2005).

Soybean is a typical short‐day (SD) plant. It undergoes reproductive growth only when the day length becomes shorter than a critical length; photoperiod‐sensitive soybean varieties can undergo flowering reversion if they are switched from SD to long‐day (LD) conditions (Han *et al*., 1997; Washburn & Thomas, 2000; Wu *et al*., 2006). This phenomenon demonstrates that soybean development is coregulated by both SD and LD effects. Using grafting experiments, Jia *et al*. (2011) revealed that some unknown floral inhibitors might be produced in leaves under LD conditions (i.e. in addition to the floral stimuli that are known to be produced under SD conditions). It is thus conceivable that some ratio of a flowering promoter and a flowering inhibitor, as specified by photoperiodic signals, may represent a sort of balance that regulates whether plants undertake vegetative growth or reproductive development (Jia *et al*., 2011).

In soybean, at least 10 *FT* homologs have been identified (Kong *et al*., 2010). *GmFT2a* and *GmFT5a* were found to be strictly photoperiod‐regulated, and have been shown to promote flowering in Arabidopsis and soybean (Kong *et al*., 2010; Sun *et al*., 2011; Cai *et al*., 2017). The functions of the other *GmFTs* in soybean remain unclear, although *GmFT2b*,* 3a*,* 3b*,* 4*,* 5b*, and *6* have been preliminarily studied using heterologous expression in Arabidopsis (Fan *et al*., 2014; Zhai *et al*., 2014; Cao *et al*., 2016a). Among the soybean *FT*s of unknown function, *GmFT4* has been noted to be strongly induced by LD conditions, and was reported to function in delaying flowering when ectopically expressed in Arabidopsis (Zhai *et al*., 2014; Cao *et al*., 2016a). Recently, it was reported that *GmFT4* appears to be the most likely candidate gene at a newly identified maturity locus, *E10* (Samanfar *et al*., 2017). Although the function of *GmFT4* still needs to be characterized, for example via overexpression in transgenic soybean, the fact that it delays flowering in Arabidopsis strongly suggests that the soybean *FT‐like* genes might have undergone functional divergence. In fact, although a majority of FT‐like proteins in various species act as floral activators (Molinero‐Rosales *et al*., 2004; Zeevaart, 2006; Komiya *et al*., 2008; Kong *et al*., 2010), functional divergence has been discovered in some species, including sugar beet (Pin *et al*., 2010), sunflower (Blackman *et al*., 2010), longan (Winterhagen *et al*., 2013), tobacco (Harig *et al*., 2012), tomato (Cao *et al*., 2016b), and onion (Lee *et al*., 2013). In this context, it is important to confirm experimentally the functions of the other *FTs* in soybean to determine whether any of them exhibit inhibitory effects.

In this study, we isolated an *FT* homolog, *GmFT1a*, from a photoperiod‐sensitive and late‐maturing soybean cv Zigongdongdou (ZGDD) that has been used as a model genotype in the study of photoperiod responses. We found that *GmFT1a* was induced in nonfloral‐inductive (i.e. LD) conditions and could delay flowering and maintain the vegetative growth of soybean, which is the complete opposite in both expression pattern and function of the known flowering promoters *GmFT2a* and *GmFT5a*. Previous work showed that the key soybean maturity gene *E1* down‐regulates the expression of *GmFT2a* and *GmFT5a* (Xia *et al*., 2012); we found here that *E1* may up‐regulate *GmFT1a* expression. Building on this, we propose a ‘teeter‐board’ model that explains what is known to date about both day‐length signals and the molecular basis of the control of vegetative and reproductive development in soybean.

## Materials and Methods

### Plant materials and growth conditions

For the expression pattern analysis of *GmFT1a*, the soybean (*Glycine max* (L.) Merr.) varieties of Zigongdongdou (ZGDD) and Heihe27, as well as several varieties from diverse maturity groups (MGs) (Supporting Information Table S1) and near‐isogenic lines (NILs) with different *E* genotypes (Wang *et al*., 2008; Table S2), were grown in a controlled culture room at 28°C under SD (12 : 12 h, light : dark), LD (16 : 8 h, light : dark), and SD13‐LD (being shifted to LD after 13 d of SD treatment) conditions (ZGDD). For the semiquantitative reverse transcription polymerase chain reaction (RT‐PCR) analysis of *GmFT1a* in the *E1* overexpression transgenic soybean lines, the soybean variety Kariyutaka and two transgenic *E1* overexpression lines with Kariyutaka background (Xia *et al*., 2012) were grown in a growth chamber at 28°C under LD conditions.

### *GmFT1a* cDNA cloning

Total RNA was extracted using Trizol (Tiangen, Beijing, China) from the trifoliolate leaves of ZGDD seedlings sampled on day 9 after commencing LD treatment; cDNA was synthesized with Superscript II reverse transcriptase (TransGen Biotech, Beijing, China) and used as a template for further *GmFT1a* cDNA cloning. Amplification was performed using PCR reactions with a KOD‐plus‐Neo DNA polymerase kit (Toyobo, Tokyo, Japan).

### Gene expression analysis

Quantitative RT‐PCR (qRT‐PCR) was performed using an ABI7900 thermocycler (Applied Biosystems, Foster City, CA, USA) with a Takara SYBR Premix Extaq (Takara, Kusatsu, Japan). Three biological replicates were analysed, with technical replicates for each of the triplicate biological samples. The qRT‐PCR data were analysed using SDS2.3 software. The primers used for real‐time quantitative PCR and the internal reference (*q‐GmActin‐F* and *q‐GmActin‐R*) are listed in Table S3. For the semiquantitative RT‐PCR analysis of *GmFT1a* in the *E1* overexpression transgenic soybean lines, fully expanded trifoliolate leaves were sampled at 4 h after dawn at 16 d after emergence (DAE) under LD conditions. The gene *TUA5* was used as a control. Twenty‐eight cycles and 32 cycles were used for the RT‐PCR analyses of *E1* and *GmFT1a*, respectively. The primers are listed in Table S3.

### Subcellular localization of GmFT1a

The open reading frame of *GmFT1a* was fused with the N‐terminus of *EGFP* under the control of the *CaMV35S* promoter. The *GmFT1a* gene was introduced into the *p16318* plasmid. Constructs were transformed into both Arabidopsis protoplast cells (Yoo *et al*., 2007) and onion epidermal cells (Liu *et al*., 2015). The GFP signal was analysed using an LSM710 confocal microscope (Zeiss, Oberkochen, Germany).

### Expression vector construction and plant transformation

A modified plasmid based on *PTF101.1* (Paz *et al*., 2006) (introducing an ‘*Asc*I’ restriction site) was used for the *35S::GmFT1a* plasmid construction: *gfp* was replaced by *GmFT1a* using the *Xba*I*‐GmFT1a‐F* and *Asc*I*‐GmFT1a‐R* primers (Table S3), so that *GmFT1a* was driven by the *CaMV35S* promoter. This plasmid was transformed into the soybean cv ‘Jack’ with the cotyledon‐node method (Paz *et al*., 2006). Plants were screened by dabbing leaves with a 160 mg l^−1^ glufosinate solution, and were genotyped for the presence of the transgene using PCR.

### Phenotyping and statistical analysis

Transgenic T_3_ plants were grown in a controlled culture room under both SD and LD conditions. We counted the number of days from emergence to the R1 stage (i.e. beginning to bloom: one open flower at any node on the main stem) and the R8 stage (full maturity: 95% of the pods had reached their mature pod color) (Fehr & Caviness, 1977). The number of additional nodes with fully expanded trifoliolate leaves (Fehr & Caviness, 1977) on the main stem produced after the R1 stage was also counted at 65 DAE under SD conditions. At least eight plants were phenotyped for each line. Data are presented as means ± SD, and Student's *t*‐tests were used to assess the significance of differences between lines.

### Transcriptome analysis and gene functional annotation

The shoot apex samples of *GmFT1a* transgenic line 10 and the wild‐type (WT) plants were collected at 16 DAE under SD conditions. Each sample consisted of material collected from five individual plants. Two biological replicates were analyzed. mRNA extracts from the samples were sequenced with the Hiseq 4000 platform (Illumina, San Diego, CA, USA) following the manufacturer's protocols. Raw data (raw reads) in the Fastq format were initially processed using in‐house Perl scripts. In this step, clean data (clean reads) were obtained by removing reads containing adapters, reads containing ploy‐N (*N* > 10%), and low‐quality reads (i.e. reads with *Q* < 5 bases for > 50% in the raw data). The clean reads were aligned to the soybean reference genome (v.275) using TopHat v.2.0.9. HTSeq v.0.5.4p3 was used to count the read numbers mapped to each gene, and then the fragments per kilobase of transcript per million mapped reads of each gene was calculated based on the length of the gene and fragments count mapped to this gene. Differential expression analysis was performed using the DESeq R package (v.1.10.1) (http://bioconductor.org/packages/2.11/bioc/html/DESeq.html). The resulting *P*‐values were adjusted using the Benjamini–Hochberg approach for controlling the false discovery rate. Genes with an adjusted *P*‐value < 0.05 found by DESeq were considered to be differentially expressed.

### Genomic cloning and sequencing

For *GmFT1a* cloning, 125 soybean varieties covering all known (14) MGs were collected (Table S4). Genomic DNA was extracted from these varieties. *GmFT1a* were amplified with a KOD‐plus‐Neo DNA polymerase kit (Toyobo) using the primers shown in Table S3. The PCR products were directly Sanger sequenced by the Tsingke Biological Technology Co. (Beijing, China). The resulting sequences were mapped to the soybean reference genome (v.Wm82.a2.v1) using BWA (v.0.7.10‐r789, using the ‘mem’ subcommand) with default settings, and *GmFT1a* polymorphisms were analyzed using Samtools (v.1.4.1, using the ‘mpileup’ subcommand) (http://samtools.sourceforge.net/) and bcftools v.1.4.1, using the ‘call’ subcommand) (https://github.com/samtools/bcftools).

### Dual‐luciferase assay

To construct the reporter vector, the 2544 bp DNA sequence upstream (5′) of the start codon of *GmFT1a* was amplified with specific primers (Table S3) from ZGDD and then subcloned into the pGreenII‐0800‐LUC vector ahead of the firefly luciferase (LUC) sequence using the *Hind*III and *BamH*I restriction sites. The effector vector was constructed by insertion of the *E1* coding DNA sequence (CDS) from ZGDD into the vector *p16318* under the control of the *CaMV35S* promoter. The reporter construct was either cotransformed with the effector construct or transformed alone into Arabidopsis protoplast cells (Yoo *et al*., 2007). A Dual‐Luciferase Reporter assay system (Promega, Madison, WI, USA) was used to measure luminescence. Relative promoter luciferase activity was calculated as the ratio of LUC : Renilla reniformis luciferase (REN). Three biological replicates were analyzed.

### Accession numbers

The sequencing data of *GmFT1a* have been deposited in NCBI under the accession numbers MG030499–MG030623. The clean data of the RNA‐seq were deposited in the SRA database of NCBI under the accession number SRP119593.

## Results

### Expression patterns of *FT* homologs during soybean flowering and flowering reversion

**Figure 1:**
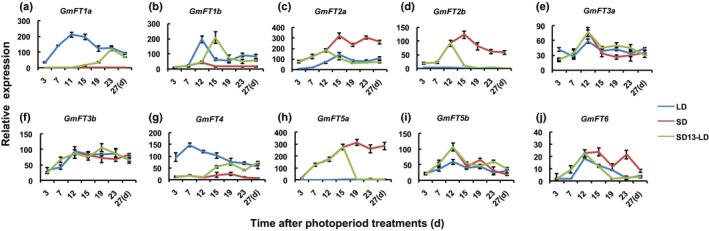
The expression of *GmFTs* during flowering reversion in soybean cv Zigongdongdou (ZGDD). (a–j) Expression profiles: (a) *GmFT1a*; (b) *GmFT1b*; (c) *GmFT2a*; (d) *GmFT2b*; (e) *GmFT3a*; (f) *GmFT3b*; (g) *GmFT4*; (h) *GmFT5a*; (i) *GmFT5b*; (j) *GmFT6*. The samples were collected 4 h after the light was turned on. The relative expression levels are normalized to *GmActin*. The data are means ± SE of three biological replicates. SD, short‐day condition (12 : 12 h, light : dark); LD, long‐day condition (16 : 8 h, light : dark); SD13‐LD, transferred to LD after 13 d of SD conditions.

We performed a qRT‐PCR analysis on the expression of *FT* family genes in ZGDD plants treated under SD, LD or SD13‐LD conditions (Li *et al*., 2005; Wu *et al*., 2006) (Fig. S1) in order to monitor the changes in expression during flowering reversion. This analysis showed that the *FT* homologs could be divided into three groups based on their expression responses to photoperiod (Fig. 1): LD‐specific genes (*GmFT1a/1b/4*), SD‐specific genes (*GmFT2a/5a/2b*), and photoperiod‐independent genes (*GmFT3a/3b/5b/6*). For the LD‐specific genes (*GmFT1a/1b/4*), the expression was barely detectable under SD conditions, but was much higher under LD conditions. After transition to LD after 13 d of SD treatment (SD13‐LD), their expression levels were rapidly increased to levels observed under LD conditions (Fig. 1a,b,g). For the SD‐specific genes (*GmFT2a/5a/2b*), the expression was much higher under SD than under LD conditions (Fig. 1c,d,h). Under SD13‐LD conditions, the expression of SD‐specific genes was dramatically reduced. For the photoperiod‐independent genes (*GmFT3a/3b/5b/6*), the expression did not show obvious fluctuations among different photoperiod conditions (Fig. 1e,f,i,j).

### Expression profiling of *GmFT1a*

**Figure 2:**
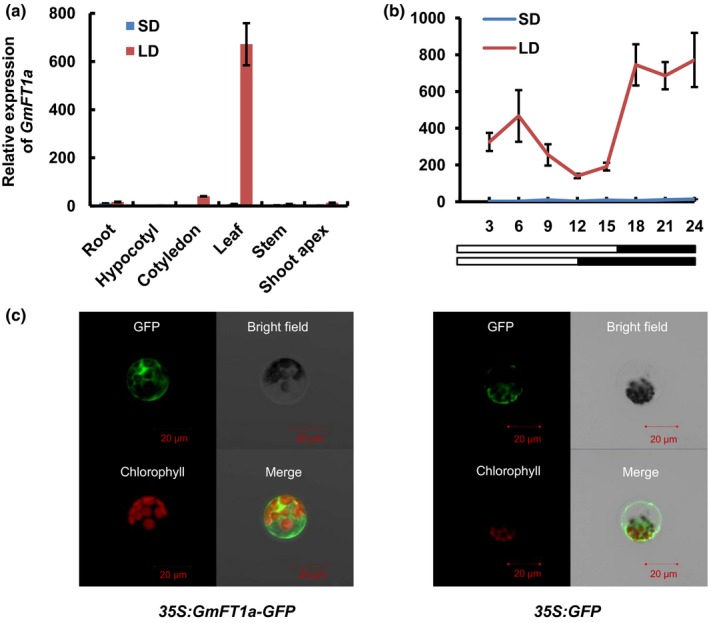
Expression pattern of *GmFT1a*. (a) Expression levels of *GmFT1a* in different organs from soybean cv Zigongdongdou (ZGDD) plants on day 9 after commencing long‐day (LD, 16 : 8 h, light : dark) or short‐day (SD, 12 : 12 h, light : dark) treatment. Samples were collected 4 h after the lights were turned on. The relative expression levels are normalized to *GmActin*. The data are means ± SE of three biological replicates. (b) Expression levels of *GmFT1a* throughout a 24 h period in unifoliate leaves of soybean cv ZGDD on day 9 of LD or SD treatment. The relative expression levels are normalized to *GmActin*. The data are means ± SE of three biological replicates. (c) Subcellular localization of a GmFT1a‐GFP fusion protein in Arabidopsis protoplasts. The left four panels are *35S:GmFT1a‐GFP* constructs, and the right four panels are controls (*35S:GFP*).

The sequence of *GmFT1a* (*Glyma.18g298900*), containing a 531 bp CDS, was cloned from the late‐maturing variety ZGDD grown under LD conditions. We examined the expression levels of *GmFT1a* in different organs (root, hypocotyl, cotyledon, leaf, stem, and shoot apex) of SD‐ and LD‐treated ZGDD plants on day 9 of the photoperiod treatments. Under LD conditions, *GmFT1a* was highly expressed in leaves, and had lower expression levels in the other organs. Under SD conditions, *GmFT1a* expression was barely detectable in any organ (Fig. 2a). We also sampled plants throughout the course of day 9 (with eight time points) to examine the diurnal expression patterns of *GmFT1a* in unifoliate leaves. Under SD conditions, barely any *GmFT1a* expression was detectable at any time (Fig. 2b). These results show that *GmFT1a* expression is induced by nonflowering‐inducing (i.e. LD) conditions.

A transient expression assay of *GmFT1a* fused to GFP was performed to ascertain the subcellular localization of GmFT1a. The constructs were transformed into Arabidopsis protoplasts (Fig. 2c) and onion epidermal cells (Fig. S2). Confocal microscopy observations suggested that the GmFT1a‐GFP fusion protein was located in the nucleus and in the cytoplasm (Figs 2c, S2), which is consistent with the subcellular localization of the other functional PEBPs in soybean (Wang *et al*., 2015).

### Overexpression of *GmFT1a* in soybean delays flowering and maturation

**Figure 3:**
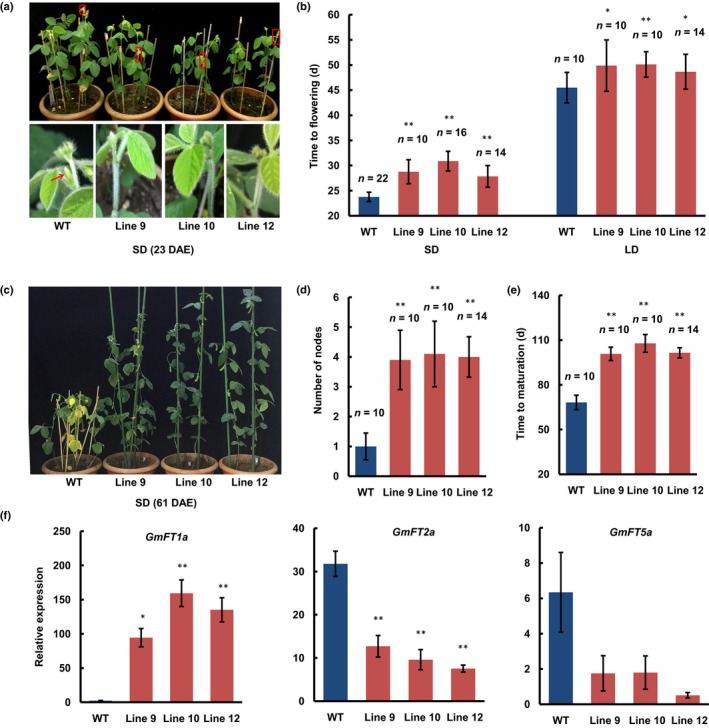
Phenotypes of the *GmFT1a* transgenic soybean plants. (a) An overview of wild‐type (WT) soybean plants (cv Jack), *GmFT1a* overexpression lines 9, 10 and 12 at 23 d after emergence (DAE) under short‐day conditions (SD, 12 : 12 h, light : dark) (upper panel), and a close‐up view of the areas framed by the boxes (lower panel). (b) Flowering times of *GmFT1a* overexpression and WT plants under SD and long‐day (LD, 16 : 8 h, light : dark) conditions. The exact numbers of individual plants are shown. The data are means ± SD, and statistical significance was determined using Student's *t*‐tests: *, *P* < 0.05; **, *P* < 0.01. (c) Overview of the WT and *GmFT1a* overexpression plants at 61 DAE under SD conditions. (d) Number of nodes in *GmFT1a* overexpression and wild‐type plants produced after R1 stage under SD conditions at 65 DAE. The exact numbers of individual plants are shown. The data are means ± SD, and statistical significance was determined using Student's *t*‐tests: **, *P* < 0.01. (e) Maturation times of *GmFT1a* overexpression and wild‐type plants under SD conditions. The exact numbers of individual plants are shown. The data are means ± SD, and statistical significance was determined using Student's *t*‐tests: **, *P* < 0.01. (f) Expression levels of *GmFT1a*,* GmFT2a*, and *GmFT5a* in leaves at 15 DAE under SD conditions. Error bars indicate the SE values of three independent plants. Statistical significance was determined using Student's *t*‐tests: *, *P* < 0.05; **, *P* < 0.01.

To investigate the function of *GmFT1a*, a construct containing the *GmFT1a* CDS driven by the *CaMV35S* promoter was transformed into the mid‐maturing soybean cv Jack. The WT plants flowered at *c*. 23.8 DAE under SD conditions. The *GmFT1a* transgenic lines flowered at 28.8 DAE (line 9), 30.9 DAE (line 10), and 27.8 DAE (line 12) (Fig. 3a,b). Under LD conditions, the flowering dates of the three transgenic lines were 4.4, 4.6, and 3.2 d later than the WT plants (Figs 3b, S3). The results of these LD and SD experiments demonstrate that the overexpression of *GmFT1a* can significantly delay soybean flowering. Furthermore, compared with WT plants, the expression of the flowering‐promoting gene *GmFT2a* was significantly lower in the leaves of the *GmFT1a* overexpression plants under SD (lines 9, 10 and 12) and LD conditions (lines 9 and 10) (Figs 3f, S3).

It is highly notable that the WT plants would stop vegetative growth *c*. 12 d after flowering, whereas the *GmFT1a* transgenic plants could maintain a much longer period of vegetative growth after flowering (Figs 3c, S4). At 65 DAE under SD conditions, the transgenic plants produced an average of 3.9 (line 9), 4.1 (line 10) and 4.0 (line 12) additional nodes after the R1 stage, while the WT plants only produced one (Fig. 3d). We tested the maturity of the WT and transgenic plants under SD conditions and found that the WT plants matured at 68.2 DAE, while plants of transgenic lines 9, 10, and 12 matured at 100.8, 107.8, and 101.5 DAE, respectively (Figs 3e, S5). These results demonstrate that in addition to its role in delaying flowering, overexpression of *GmFT1a* can significantly delay soybean maturation.

### Differentially expressed genes in the *GmFT1a* overexpression plants

**Figure 4:**
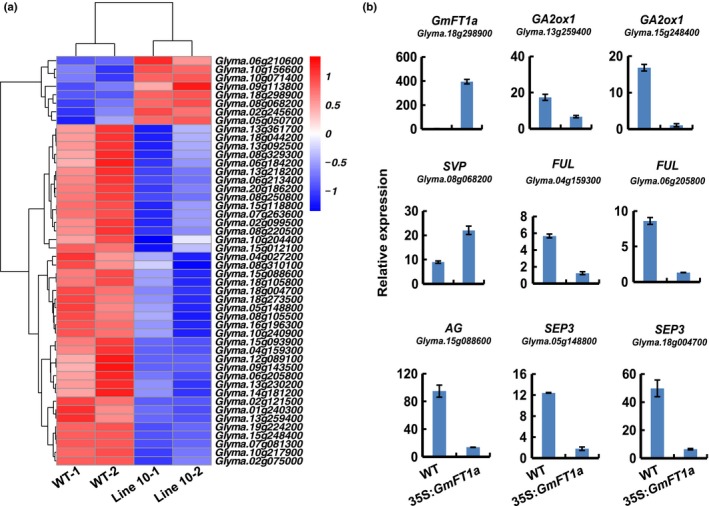
Transcriptome analysis of transgenic soybean plants overexpressing *GmFT1a*. (a) Differentially expressed genes (DEGs) associated with regulation of flowering time between *GmFT1a* overexpression line 10 and wild‐type plants. Red indicates up‐regulated genes and blue indicates down‐regulated genes. (b) Validation of the expression of selected flowering‐related DEGs by quantitative reverse transcription polymerase chain reaction. The relative expression levels are normalized to *GmActin*. The data are means ± SE of three biological replicates. WT, wild type soybean (cv Jack).

We used transcriptome sequencing (RNA‐Seq) of the WT and *GmFT1a* transgenic plants (line 10) to explore differential gene expression in response to overexpression of *GmFT1a*. Compared with the WT, there were 3120 differentially expressed genes (DEGs) in the *GmFT1a* transgenic plants (Table S5). Comparative analysis using the phytozome and Uniprot databases indicated that at least 48 overlapping DEGs (including *GmFT1a*) showed homology with known flowering time‐associated genes from Arabidopsis. Several DEGs were involved in photoperiod, GA, trehalose‐6‐phosphate (T6P), or sugar signaling pathways (Fig. 4a; Table S6). *Glyma.19g224200*, which is a homolog of *Arabidopsis thaliana Phytochrome A* (*PHYA*) (Mockler *et al*., 2003), was down‐regulated. Four *TEMPRANILLO 1* (*TEM1*) (Castillejo & Pelaz, 2008) homologs (*Glyma.02g099500*,* Glyma.01g240300*,* Glyma.10g204400*,* Glyma.20g186200*) were down‐regulated, and one (*Glyma.10g156600*) was up‐regulated. The homologs of GA pathway genes, including *GA2oxidase1* (*Glyma.13g259400*,* Glyma.15g248400*), *GA2oxidase2* (*Glyma.13g218200*), *GA3oxidase2* (*Glyma.15g012100*,* Glyma.13g361700*), and *GA20oxidase2* (*Glyma.15g093900*) were all down‐regulated. Two T6P synthase homologs (*Glyma.06g184200* and *Glyma.13g092500*), along with two sucrose metabolism homologs (*Glyma.10g217900*,* Glyma.02g075000*), were also down‐regulated. Notably, the flowering repressor genes *SHORT VEGETATIVE PHASE* (*SVP)* (Andrés *et al*., 2014) (*Glyma.08g068200*) (Jung *et al*., 2012) and *TERMINAL FLOWER1* (*TFL1*) (Hanzawa *et al*., 2005) (*Glyma.10g071400*) were up‐regulated, but two flowering promoter gene *FRUITFULL* (*FUL*) homologs (Balanzà *et al*., 2014) (*GmFULa*:* Glyma.06g205800*;* GmFULb: Glyma.04g159300*) (Jia *et al*., 2015) were down‐regulated. The expression of genes associated with floral meristem identity, including *AGAMOUS* (*AG*) (Favaro *et al*., 2003) (*Glyma.15g088600*), *SEPALLATA 1* (*SEP1*) (*Glyma.08g250700*,* Glyma.18g273500*), *SEPALLATA 3* (*SEP3*) (*Glyma.05g148800*,* Glyma.08g105500*,* Glyma.10g240900*,* Glyma.18g004700*), *SEPALLATA 4* (*SEP4*) (*Glyma.02g121500*) (Ma *et al*., 1991) and *APETALA1* (*AP1*) (Simon *et al*., 1996) (*GmAP1b*:* Glyma.01g064200*;* GmAP1c*:* Glyma.08g250800*) (Nan *et al*., 2014) were all down‐regulated (Fig. 4a; Table S6). We then performed qRT‐PCR assays with shoot apex samples to verify the expression of nine genes identified from our RNA‐Seq analysis; the results suggested that the expression of these genes were consistent with the RNA‐Seq results (Fig. 4b).

### Haplotype analysis of *GmFT1a*

We cloned the genomic loci of *GmFT1a* in 125 soybean varieties covering all 14 MGs (Table S4). Six missense polymorphism sites and one stop gained site were found (Table S7). Based on these data, eight haplotypes were clustered (Table S7). Of these haplotypes, HT8 predominated among the sequenced varieties. It is notable that the HT1 and HT4 hapolotypes were only found, respectively, in MGs later than MG 0 and MG II (Table S7) (Fig. S6).

### *GmFT1a* expression is related to maturity in soybean

**Figure 5:**
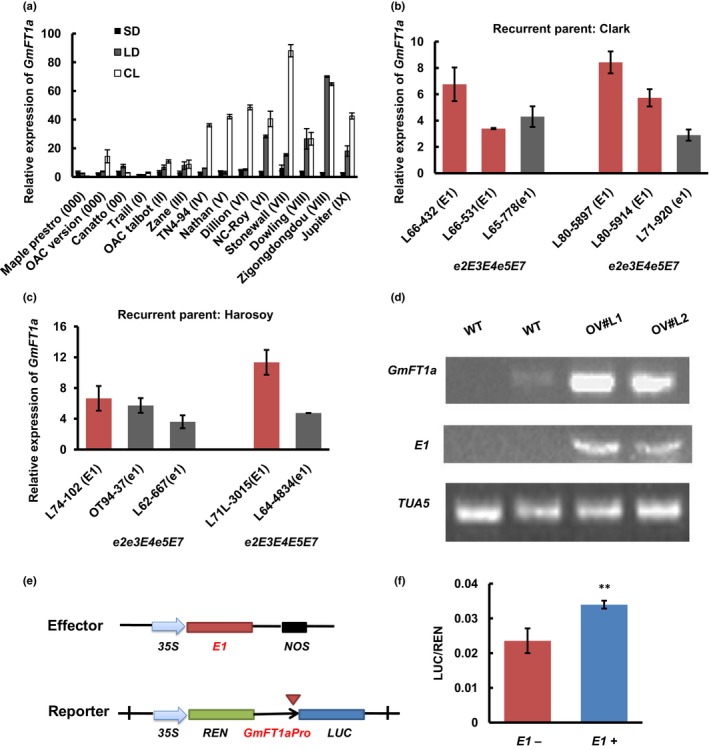
The expression of *GmFT1a* affects soybean maturity and may be positively regulated by *E1*. (a) Expression of *GmFT1a* in 14 varieties (from different maturity groups of soybean) on day 9 of the different photoperiod treatments. The numbers in the bracket after each variety name denote the maturity group of each variety. Samples (unifoliate leaves) were collected 4 h after the light was turned on. The data are means ± SE of three independent experiments. SD, short day (12 : 12 h, light : dark); LD, long day (16 : 8 h, light : dark); CL, continuous light (24 h light). (b, c) Expression of *GmFT1a* in near‐isogenic lines (NILs) derived from soybean varieties of Clark (b) and Harosoy (c) under LD conditions. Fully expanded unifoliate leaves were collected 4 h after the light was turned on (9 d after emergence, DAE) under LD conditions. The relative expression levels are normalized to *GmActin*. The data are means ± SE of three independent experiments. (d) Semiquantitative reverse transcription polymerase chain reaction analysis of *GmFT1a* in the wild‐type (WT) soybean plants (cv Kariyutaka) and *E1* overexpression transgenic soybean lines OV#L1 and OV#L2. Fully expanded trifoliolate leaves were sampled 4 h after the light was turned on (16 DAE) under the LD condition. (e) Schematic representation of effector and reporter constructs used in the transient expression assays. (f) Quantitation of relative reporter activities. LUC (luciferase) activities, normalized to Renilla luciferase (REN) activity, are defined as relative expression units (REU). Data (mean ± SD) presented are representative of three technical replicates for three biological replicates, and statistical significance was determined using Student's *t*‐tests: **, *P *< 0.01.

We analyzed the expression of *GmFT1a* in Heihe27 (Jia *et al*., 2011), a photoperiod‐insensitive and early‐flowering variety. *GmFT1a* expression was not detected in this variety under SD or LD conditions, highlighting a very different expression pattern for this gene than the pattern observed in the late‐flowering variety, ZGDD (Fig. S7). We then analyzed *GmFT1a* expression in several North American varieties from different MGs (Jiang *et al*., 2014) on day 9 after commencing photoperiod treatments (SD, LD, and continuous light (CL); Table S1). The expression of *GmFT1a* was very low in these varieties under SD conditions. Under LD conditions, *GmFT1a* expression levels were also low in early‐maturing varieties, but were noticeably higher in the varieties belonging to the MG VI‐IX. Under CL (24 h light) conditions, high levels of *GmFT1a* expression were detected in varieties belonging to MG IV‐IX (Fig. 5a).

The *E1* gene, which was specifically expressed in plants grown under LD conditions, delays soybean flowering and has a very strong effect on maturation time (Xia *et al*., 2012). To determine whether *E1* affects *GmFT1a* expression, we examined *GmFT1a* expression in the maturity gene (*E*) NILs derived from Clark and Harosoy (Table S2) (Bernard *et al*., 1991; Wang *et al*., 2008). The results showed that a majority of the NILs with the dominant genotype (*E1*) (except L66‐531) had relatively higher *GmFT1a* expression compared with NILs with the recessive genotype (*e1*) (Fig. 5b,c). Note that this is very similar to the expression pattern for *GmFT4* (Fig. S8), a putative flowering repressor in soybean (Zhai *et al*., 2014). Moreover, in the *E1*‐overexpressed transgenic soybean lines, the *GmFT1a* expression pattern is also highly similar to that of *GmFT4*. Namely, the expression of *GmFT1a* was elevated compared with that in WT (Fig. 5d).

We next examined whether *E1* regulates the *GmFT1a* promoter by using a dual‐luciferase assay. In this experiment, Arabidopsis protoplast cells were cotransformed with a plasmid expressing a dual‐luciferase reporter driven by the *GmFT1a* promoter and a plasmid expressing *E1* (Fig. 5e). Expression of the REN driven by the standard *CaMV35S* promoter was used as the internal control. The experiment demonstrated that the presence of the E1 protein significantly increased the levels of LUC activity under the control of *GmFT1a* promoter (Fig. 5f; 1.44‐fold increase), suggesting that *E1* may up‐regulate *GmFT1a* expression.

## Discussion

### Functional diversification of FT in soybean

Studies in Arabidopsis have established that the FT protein promotes flowering, yet some *FT* homologs in other species have recently been found to have inhibitory effects on flowering (Blackman *et al*., 2010; Pin *et al*., 2010; Harig *et al*., 2012; Lee *et al*., 2013; Winterhagen *et al*., 2013). FT homologs thus seem to have diverged in function.

Soybean has undergone two whole‐genome duplication events which have resulted in multiple copies of homologs in soybean that are only single‐copy genes in Arabidopsis (Schmutz *et al*., 2010). Among the 10 *FT* homologs in soybean, *GmFT2a* and *GmFT5a* have been reported as possible candidates for florigen induced by SD conditions (Kong *et al*., 2010; Sun *et al*., 2011). However, in this study, we reported that *GmFT1a*, an *FT* homolog in soybean, is induced by LD conditions (Figs 1a, 2) and significantly inhibits flowering and maturation (Fig. 3). This finding provides clear evidence that the *FT* homologs have functionally diverged in soybean. GmFT1a can thus be viewed as a candidate for ‘antiflorigen’, which has been proposed to be produced in leaves under noninductive photoperiod conditions and to antagonize florigen (Matsoukas, 2015).

In soybean production, the vegetative growth periods of temperate varieties are shortened when they are grown in tropical and subtropical areas, resulting in low yields (Hartwig, 1970). However, the long‐juvenile varieties have higher yields because of a longer vegetative period and greater biomass accumulation. Like the mutated allele of *J* (*GmELF3*) (Lu *et al*., 2017; Yue *et al*., 2017), *GmFT1a* might contribute to delaying the flowering time of soybean varieties, and could be used to ensure the successful deployment of high‐yield germplasm in tropical environments (Hartwig & Kiihl, 1979).

### *GmFT1a*‐mediated changes in the expression of flowering‐related genes in the shoot apex under SD conditions

By analyzing the transcriptome of the *GmFT1a* transgenic plants, we identified several flowering‐related genes that respond to the overexpression of *GmFT1a* in soybean. In Arabidopsis, the flowering inhibitor *SVP* regulates flowering by down‐regulating the expression of *SEP3* and *GA20ox2*, a GA biosynthesis gene (Andrés *et al*., 2014). Moreover, *FUL*, a MADS‐box transcription factor, is induced by FT in the shoot apical meristem, and functions in multiple developmental processes such as floral meristem identity specification, shoot maturation, and the control of floral transition (Hempel *et al*., 1997; Gu *et al*., 1998; Ferrándiz *et al*., 2000; Balanzà *et al*., 2014). During floral initiation in soybean, the expression of *FUL* and *SEP3* increases, indicating their potential roles in the floral transition (Wong *et al*., 2009). In our RNA‐Seq study, the expression patterns of these genes in soybean (*SVP*,* SEP3*,* GA20 ox2*, and *FUL*) changed in response to overexpression of *GmFT1a* (Fig. 4). Although the functions of these genes in soybean remain unclear, they may act as the downstream genes of *GmFT1a* and contribute to flowering transition.

We found that the overexpression of *GmFT1a* down‐regulates the expression of the floral organ identity specification genes *GmAP1b* and *GmAP1c*. This is the complete opposite of the regulation pattern that was observed in a study by Nan *et al*. (2014), in which plants overexpressing flowering‐promoting genes (*GmFT2a*,* GmFT5a*) had increased expression of the floral organ identity specification gene *GmAP1s*. Collectively, these findings indicate that the flowering promoter GmFT2a/5a and flowering inhibitor GmFT1a may regulate the same set of downstream genes in some as‐yet‐unclear competitive/antagonistic manner. To help clarify this, future studies should focus on whether these FT homologs are transmissible and should seek to determine whether or not they act with other known regulators of flowering (e.g. FD) in the shoot apex.

### A possible model for specifying either vegetative or reproductive development in soybean

**Figure 6:**
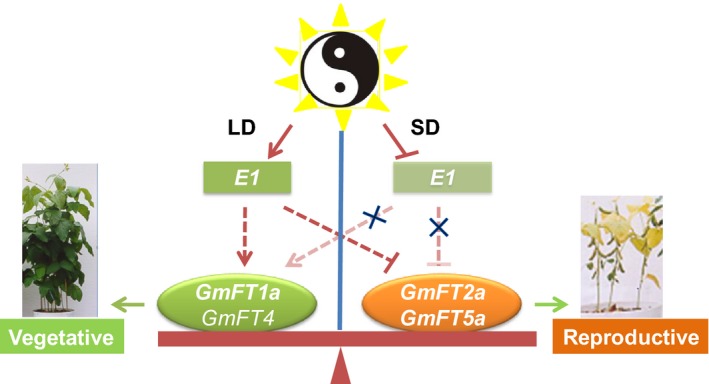
A teeter‐board model for flowering time regulation in soybean. Red arrows represent stimulation of gene expression. Red T‐shaped symbols represent inhibition of gene expression; green arrows represent the promoting effects on plant development. The X symbols represent the negation of inhibition/promotion. The dotted arrow and dotted T‐shaped symbol represent the stimulation or inhibition effect, which remains to be experimentally confirmed. ‘*GmFT4*’ is in a nonbold font to indicate that its function has not been fully confirmed experimentally. ‘*E1*’ is in a translucent box to indicate that its expression is inhibited under SD conditions. The translucent arrow or T‐shaped symbol indicates that the promotion/inhibition effect is weakened. The red box represents the proposed teeter board. SD, short‐day condition (12 : 12 h, light : dark); LD, long‐day condition (16 : 8 h, light : dark).

*E1*, a key soybean maturity gene, is induced by LD conditions and inhibits flowering and maturation in soybean (Xia *et al*., 2012). Similar to the putative flowering inhibitor *GmFT4* (Zhai *et al*., 2014), but the opposite of the reported flowering promoters, *GmFT2a* and *GmFT5a* (Kong *et al*., 2010; Sun *et al*., 2011; Xia *et al*., 2012), the flowering inhibitor *GmFT1a*, which is induced under LD conditions, appears to be up‐regulated by *E1*. Based on the results of the present study and previous reports (Kong *et al*., 2010; Sun *et al*., 2011; Xia *et al*., 2012; Zhai *et al*., 2014), we propose a ‘teeter‐board’ model for the specification of either vegetative or reproductive development in soybean (Fig. 6). In this model, *E1* acts as a photoperiod‐dependent switch that can up‐regulate the expression of the flowering‐inhibiting genes *GmFT1a* but down‐regulate the expression of the flowering‐promoting genes *GmFT2a* and *GmFT5a*. Leaves sense day length and regulate the amount of floral promoters (GmFT2a/GmFT5a) and inhibitors (GmFT1a and possibly GmFT4) by switching the expression of *E1*, consequently determining the direction of soybean development.

## Author contributions

W.L. performed the vector construction and subcellular location assays. W.L. and B.J. performed the expression analysis and dual‐luciferase assay; B.J., L.M. and S.Z. performed the gene cloning, and the transcriptome and haplotype analyses; H.Z. and Z.X. analyzed *GmFT1a* expression in *E1* overexpression soybean transformants; X.X. assisted in the analysis of gene expression during soybean flowering and flowering reversion; C.W. and S.S. performed the phenotype observations and measurements; T.W. participated in the data analysis; W.H. and L.C. participated in the soybean transformation; T.H. conceived the research. W.L., B.J. and T.H. wrote the manuscript.

## Supporting information

Please note: Wiley Blackwell are not responsible for the content or functionality of any Supporting Information supplied by the authors. Any queries (other than missing material) should be directed to the *New Phytologist* Central Office.

**Fig. S1** Phenotypes observed in soybean cv Zigongdongdou (ZGDD) with different photoperiod treatments.
**Fig. S2** Subcellular localization of a GmFT1a‐GFP fusion protein in onion epidermal cells.
**Fig. S3** The *GmFT1a* overexpression soybean plants exhibit late flowering under LD conditions.
**Fig. S4** The *GmFT1a* overexpression soybean plants maintain vegetative growth longer under SD conditions.
**Fig. S5** The phenotype of *GmFT1a* overexpression soybean line 10 under SD at 65 DAE.
**Fig. S6** The distribution of *GmFT1a* haplotypes (HT) in soybean varieties differing in maturity groups (MGs).
**Fig. S7** *GmFT1a* expression levels in the leaves of soybean varieties of Zigongdongdou (ZGDD) and Heihe27 under LD and SD conditions.
**Fig. S8** Expression of *GmFT4* in the near‐isogenic lines (NILs) derived from soybean varieties of Clark and Harosoy under LD conditions.
**Table S1** Soybean varieties from North America and their respective maturity groups
**Table S2** The soybean near‐isogenic lines (NILs) in this study and their *E* genotypes
**Table S3** Sequences of primers used in this study

**Table S4** Soybean varieties for the sequencing analysis of *GmFT1a* genomic loci
**Table S5** Differentially expressed genes in plants of *GmFT1a* overexpression soybean line 10
**Table S6** Differentially expressed flowering‐regulation associated genes in plants of *GmFT1a* overexpression soybean line 10
**Table S7** *GmFT1a* haplotypes (HT) in soybean varieties differing in maturity groups (MGs)
