# Supplementary material for: Functional diversification of Flowering Locus T homologs in soybean: GmFT1a and GmFT2a/5a have opposite roles in controlling flowering and maturation
Source: New Phytol. 2017 Nov 9;217(3):1335–45. doi: 10.1111/nph.14884 (PMC5900889; doi:10.1111/nph.14884)
Supplement: Supplementary file 1 — Fig. S1 Phenotypes observed in soybean cv Zigongdongdou (ZGDD) with different photoperiod treatments. Fig. S2 Subcellular localization of a GmFT1a‐GFP fusion protein in onion epidermal cells. Fig. S3 The GmFT1a overexpression soybean plants exhibit late flowering under LD conditions. Fig. S4 The GmFT1a overexpression soybean plants maintain vegetative growth longer under SD conditions. Fig. S5 The phenotype of GmFT1a overexpression soybean line 10 under SD at 65 DAE. Fig. S6 The distribution of GmFT1a haplotypes (HT) in soybean varieties differing in maturity groups (MGs). Fig. S7 GmFT1a expression levels in the leaves of soybean varieties of Zigongdongdou (ZGDD) and Heihe27 under LD and SD conditions. Fig. S8 Expression of GmFT4 in the near‐isogenic lines (NILs) derived from soybean varieties of Clark and Harosoy under LD conditions. Table S1 Soybean varieties from North America and their respective maturity groups Table S2 The soybean near‐isogenic lines (NILs) in this study and their E genotypes Table S3 Sequences of primers used in this study [file NPH-217-1335-s001.pdf]

New Phytologist Supporting Information Figs S1–S8 and Tables S1–S3

Article title: Functional diversification of *Flowering Locus T* homologs in soybean: *GmFT1a* and *GmFT2a/5a* have opposite roles in controlling flowering and maturation

Authors: Wei Liu, Bingjun Jiang, Liming Ma, Shouwei Zhang, Hong Zhai, Xin Xu, Wensheng Hou, Zhengjun Xia, Cunxiang Wu, Shi Sun, Tingting Wu, Li Chen and Tianfu Han

Article acceptance date: 3 October 2017

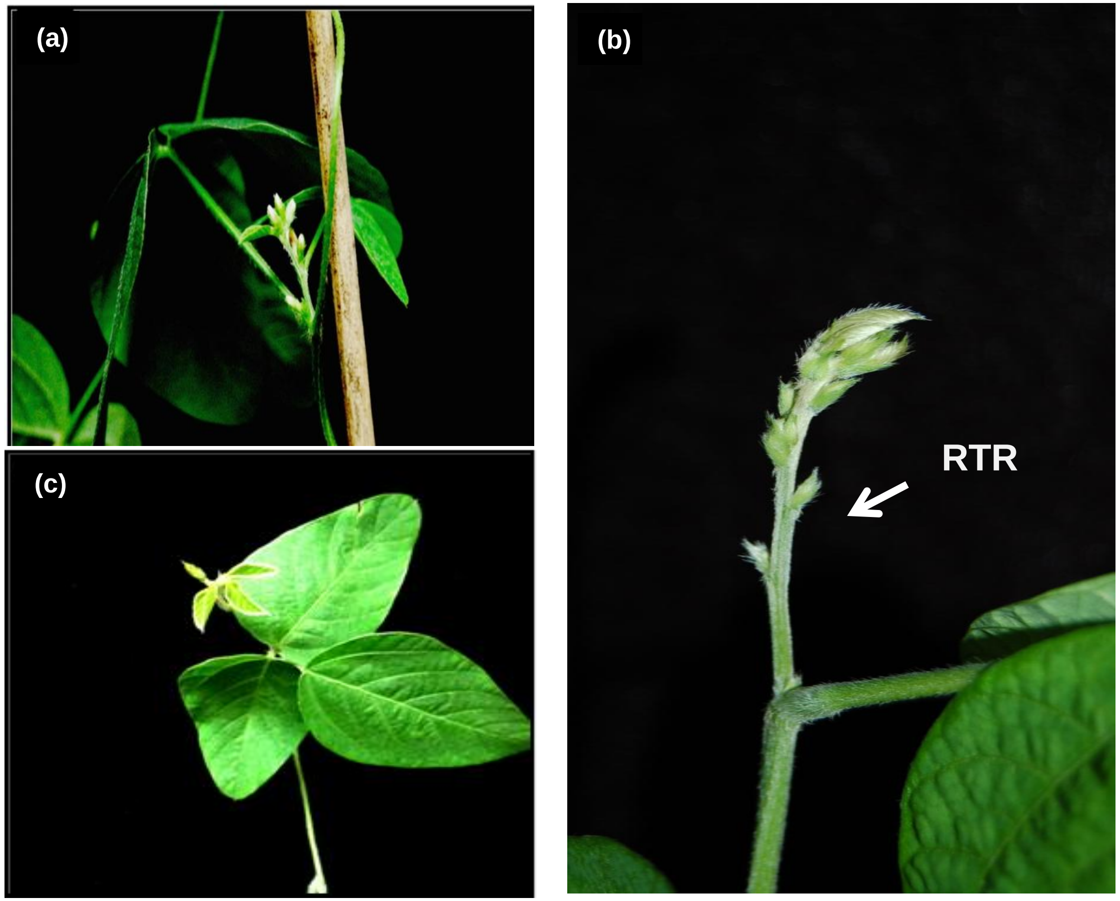

Fig. S1 Phenotypes observed in soybean variety Zigongdongdou (ZGDD) with different photoperiod treatments.

(a) ZGDD flowered on the 30th day under SD conditions. (b) Example of flower reversion (image taken 39 days after transfer from SD to LD). (c) The apical meristem maintained vegetative growth under LD. RTR: reversed terminal raceme. SD: Short day condition (12/12 h light/dark); LD: Long day condition (16/8 h light/dark)

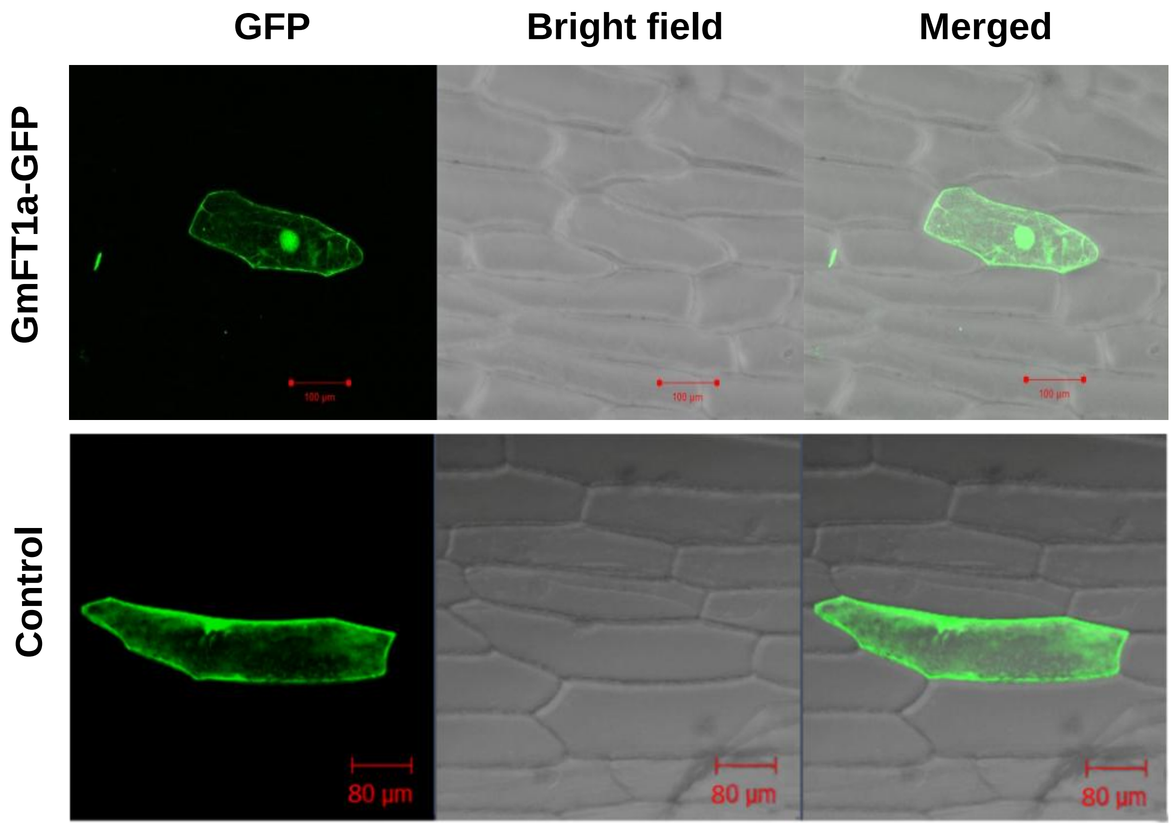

Fig. S2 Subcellular localization of a GmFT1a-GFP fusion protein in onion epidermal cells. The upper three panels are *35S:GmFT1a-GFP* constructs and the lower three panels are *35S:GFP* controls.

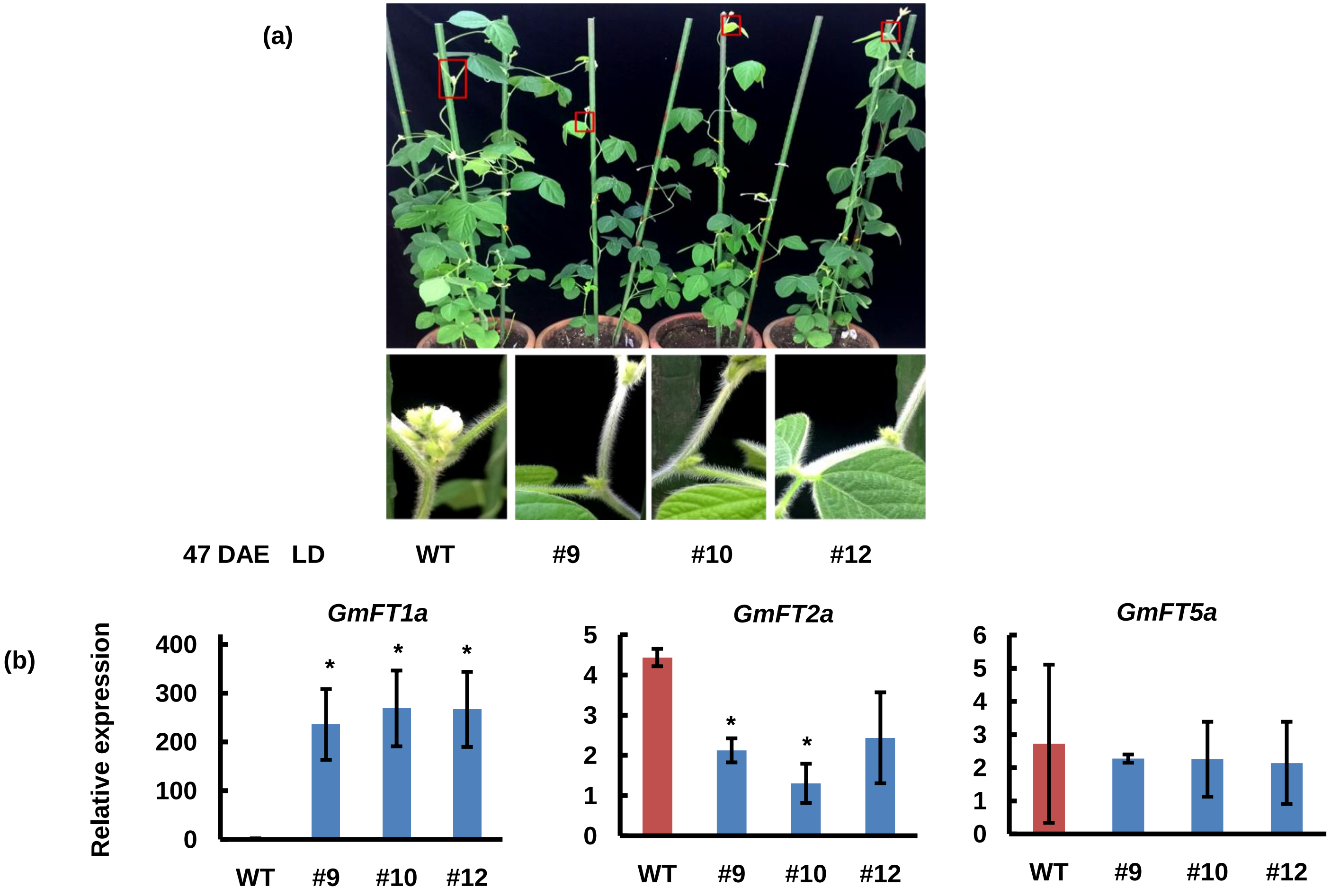

Fig. S3 The *GmFT1a* overexpression soybean plants exhibit late flowering under LD.

(a) The phenotype of wild-type (WT) soybean plants (variety of Jack) and the late flowering *GmFT1a* overexpression plants at 47 DAE under LD. LD: 16/8 h light/dark. (b) The expression levels of *GmFT1a*, *GmFT2a*, and *GmFT5a* in leaves (40 DAE) under LD. Error bars indicate the SE (standard error) values of three independent plants. Statistical significance was determined using Student's *t*-tests (* $p<0.05$).

DAE: days after emergence.

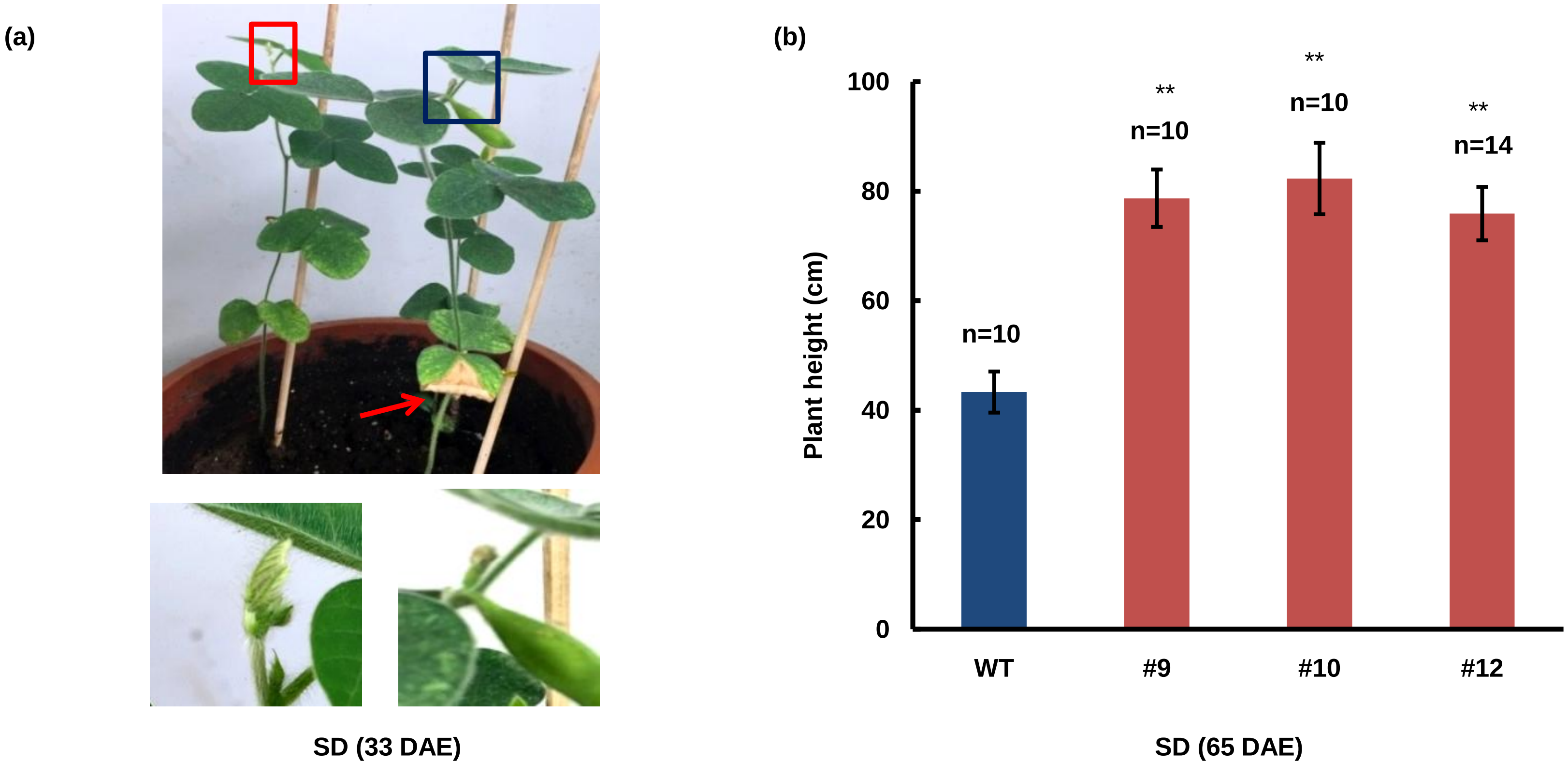

Fig. S4 The *GmFT1a* overexpression soybean plants maintain vegetative growth longer under SD. (a) The phenotype of plants of *GmFT1a* overexpression line #10 under SD (33 DAE). The upper line is an overview of transgenic line plants, #10. The red arrow indicates a plant lacking resistance to glufosinate. The lower line is a zoomed-in view of the areas framed by the red box (glufosinate-resistant plant) and blue box (plant lacking resistance to glufosinate). The shoot apex of positive *GmFT1a* transgenic plants that are still in the vegetative growth. The shoot apex of a plant lacking resistance to glufosinate transitioned into reproductive growth. (b) The height for wild-type and *GmFT1a* overexpression plants at 65 DAE under SD. The data represent the mean ± standard deviation, and statistical significance was determined using Student's *t*-tests (** $p<0.01$).SD: Short day condition (12/12 h light/dark); DAE: days after emergence.

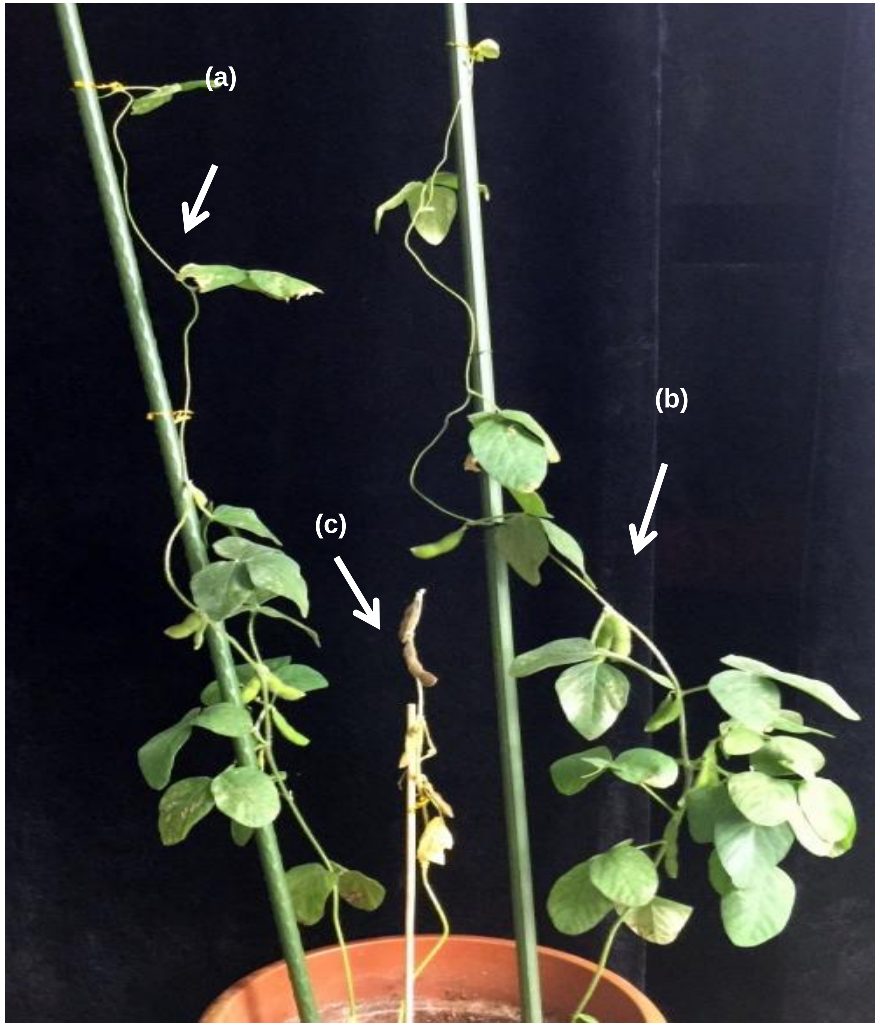

Fig. S5 The phenotype of the soybean plants of *GmFT1a* overexpression soybean line #10 under SD at 65 DAE.

(a) and (b) two positive *GmFT1a* overexpression plants; (c) a plant that has no resistance to glufosinate. SD: 12/12 h light/dark; DAE: days after emergence.

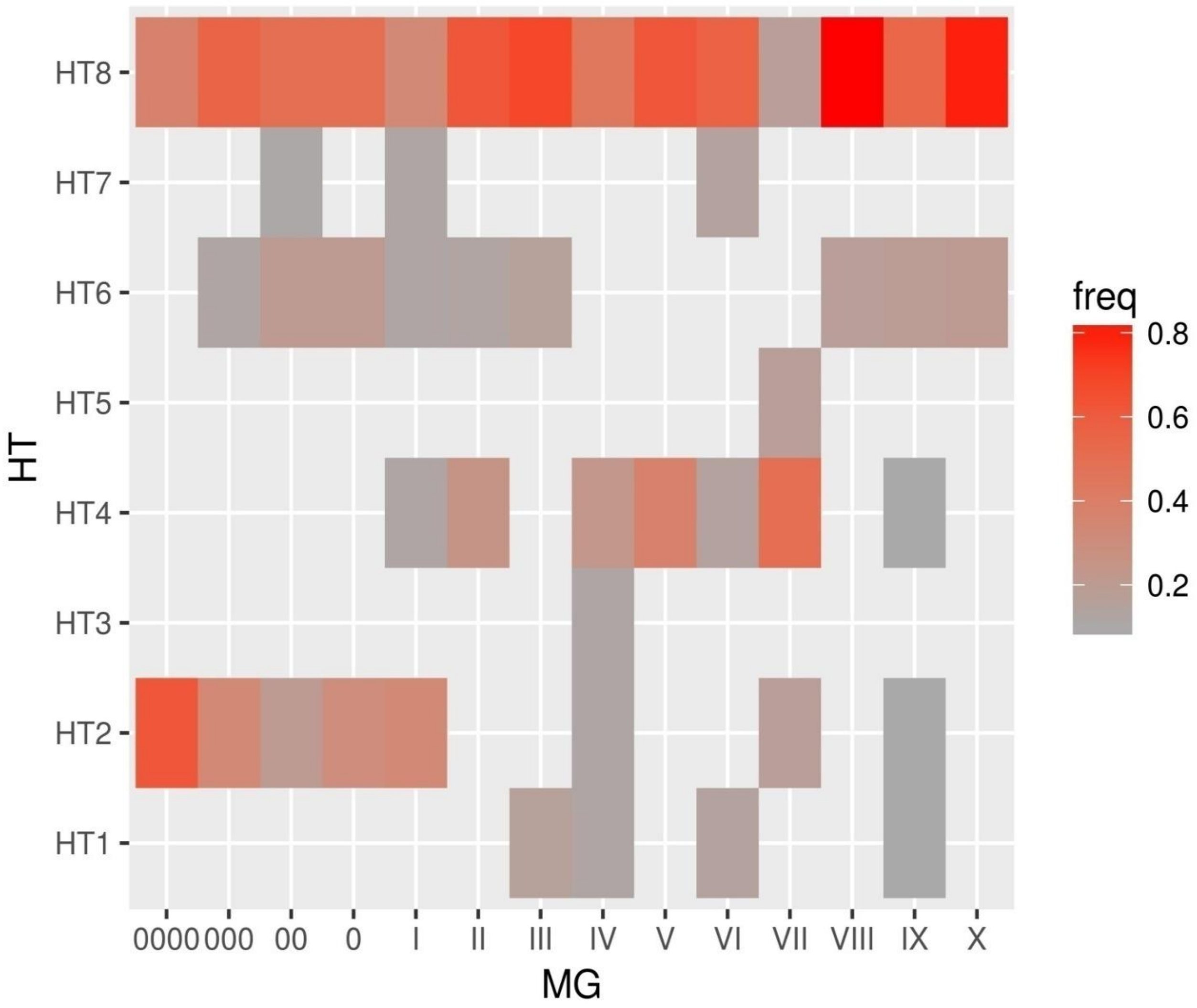

Fig. S6 The distribution of *GmFT1a* haplotypes (HT) in soybean varieties differing in maturity groups (MG).

HT1-HT8 denote *GmFT1a* haplotypes. The numbers on the X axis denote the maturity group. Freq means *GmFT1a* haplotype frequency in each maturity group.

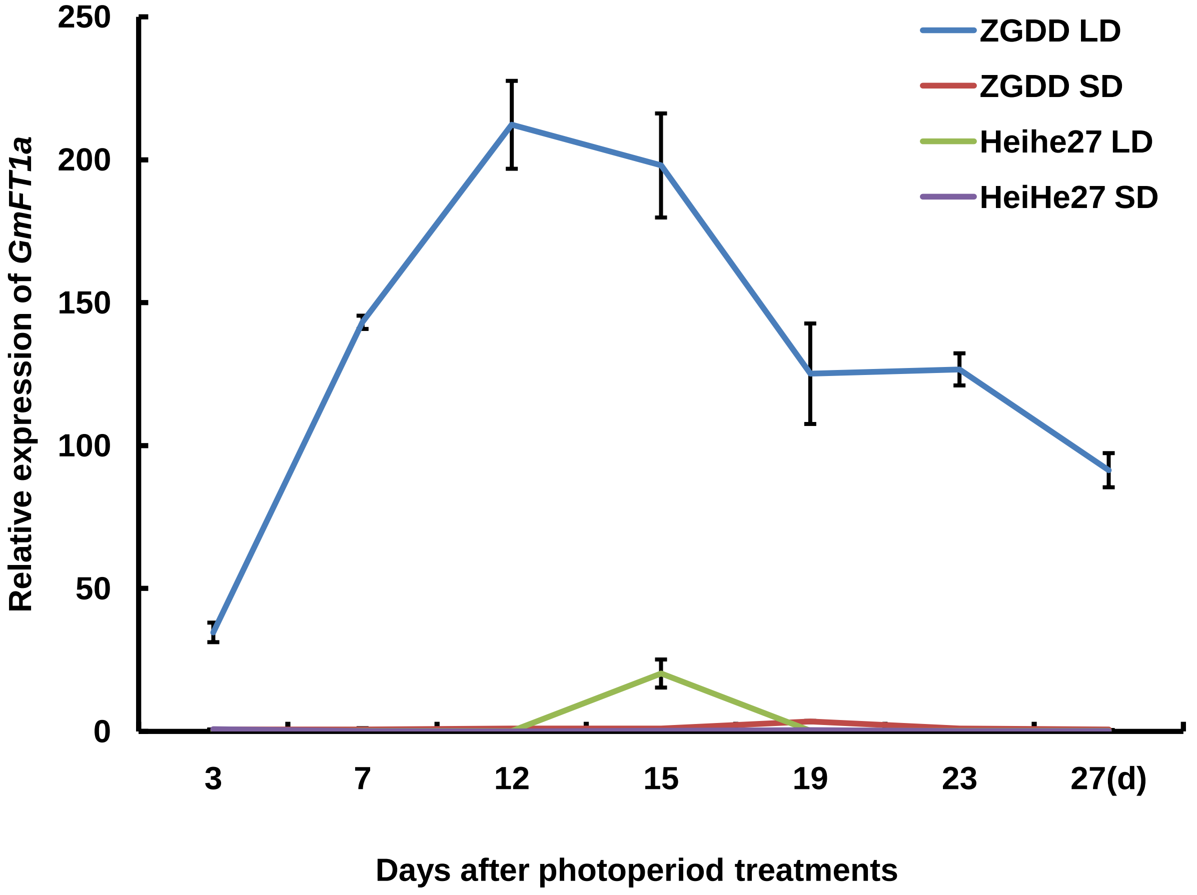

Fig. S7 *GmFT1a* expression levels in the leaves of soybean varieties of Zigongdongdou (ZGDD) and Heihe27 under LD and SD. The relative expression levels are normalized to *GmActin*. The data represent the mean ± SE (standard error) of three independent experiments. LD: 16/8 h light/dark; SD: 12/12 h light/dark.

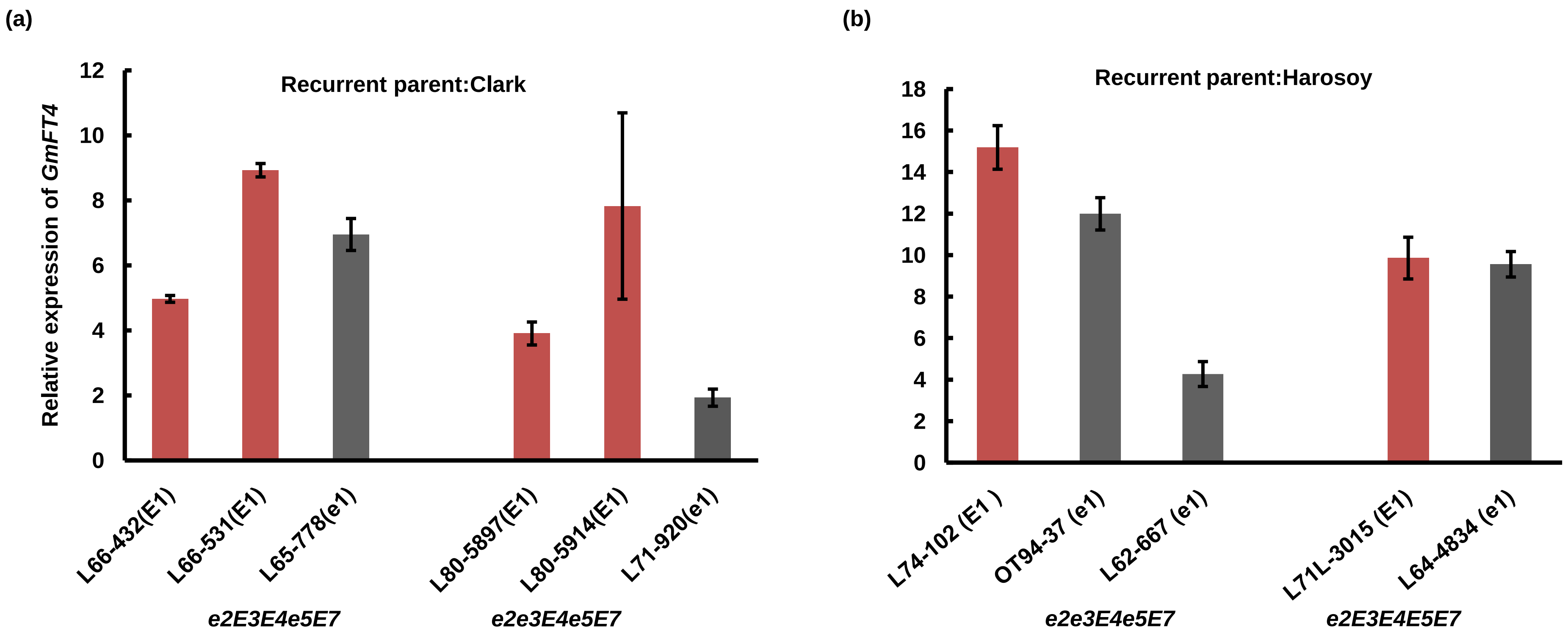

Fig. S8 Expression of *GmFT4* in the near-isogenic lines (NILs) derived from soybean varieties of Clark (a) and Harosoy (b) under LD condition. Fully expanded unifoliate leaves were collected 4 h after the light was turned on (9 DAE) under LD conditions. The relative expression levels are normalized to *GmActin*. The data represent the mean ± SE (standard error) of three independent experiments. LD: 16/8 h light/dark; DAE: days after emergence.

Table S1. Soybean varieties from North America and their respective Maturity Groups

Variety	PI number	Maturity Group (MG)	Genotype[1]
Maple Prestro	PI548594	000	*e1e2e3e4*
OAC Version	PI567787	000	*e1e2e3e4*
Canatto	PI548648	00	*e1-ase2e3e4*
Traill	PI596541	0	*e1-ase2e3E4*
OAC Talbot	PI567786	II	*e1-ase2E3E4*
Zane	PI548634	III	*e1-asE2E3E4*
TN4-94	PI598222	IV	*E1E2E3E4*
Nathan	PI564849	V	*E1E2E3E4*
Dillon	PI592756	VI	*E1E2E3E4*
NC-Roy	PI617045	VI	*E1E2E3E4*
Stonewall	PI531068	VII	*E1E2E3E4*
Dowling	PI548663	VIII	*E1E2E3E4*
Jupiter	PI548972	IX	*E1E2E3E4*

[1] **Jiang B, Nan H, Gao Y, et al. 2014**. Allelic combinations of soybean maturity loci E1, E2, E3 and E4 result in diversity of maturity and adaptation to different latitudes. *PloS One* **9**: e106042.

Table S2 The soybean near-isogenic lines (NILs) in this study and their *E* genotypes

NIL	Genotype	Recurrent parent
L66-531	*E1e2E3E4e5E7*	Clark
L66-432	*E1e2E3E4e5E7*	Clark
L65-778	*e1e2E3E4e5E7*	Clark
L80-5897	*E1e2e3E4e5E7*	Clark
L80-5914	*E1e2e3E4e5E7*	Clark
L71-920	*e1e2e3E4e5E7*	Clark
L74-102	*E1e2e3E4e5E7*	Harosoy
OT94-37	*e1e2e3E4e5E7*	Harosoy
L62-667	*e1e2e3E4e5E7*	Harosoy
L71L-3015	*E1e2E3E4E5E7*	Harosoy
L64-4834	*e1e2E3E4E5E7*	Harosoy

Table S3 Sequences of primers used in this study

Primer Name	Primer Sequence
**For *GmFT1a CDS* cloning**	
GmFT1a- F	5′-ATGCCTAGATCAACGGACCCTCT -3′
GmFT1a- R	5′-TTATCTTCTTCTTCCACTGCTT -3′
**For Construction of 35S::*GmFT1a-GFP* and 35S::*GmFT1a***	
Xba I-*GmFT1a-F*	5′-TCTAGAATGCCTAGATCAACGGACCCTCT -3′
Sal I-*GmFT1a-R*	5′-ACGCGT TTATCTTCTTCTTCCACTGCTT -3′
Asc I-*GmFT1a-R*	5′-GGCGCGCC TTATCTTCTTCTTCCACTGCTT-3′
**For Real-Time Quantitative PCR**	
qGmFT1a -F	5′-TGCAACTACAGGGGCAAACT-3′
qGmFT1a-R	5′-GTTGAAGTACATGGCCGCGA-3′
qGmFT1b-F	5′-CAGCAAACGTACGCAAGAGC-3
qGmFT1b-R	5′-AACTTCGGAGCAGCTGTCAT-3
qGmFT2a-F	5′-GGATTGCCAGTTGCTGCTGT -3′
qGmFT2a-R	5′-GAGTGTGGGAGATTGCCAAT -3′
qGmFT2b-F	5′-ATGCCTCGTGGAAGTAGGGA -3′
qGmFT2b-R	5′-CACTTGGGCTAGGTGCATCA-3′
qGmFT3a-F	5′-GGGTGTATGCTCCTGGATGG-3′
qGmFT3a-R	5′-CACCAGAGCCAGTTTCCCTC-3′
qGmFT3b-F	5′-GGGGATTCATCGGTTGGTGT-3′
qGmFT3b-R	5′-CACCAGAGCCACTTTCCCTC-3′
qGmFT5a-F	5′-GCCTTACTCCAGCTTATACT-3′
qGmFT5a-R	5′-GGCATGCTCTAGCATTGCAA-3′
qGmFT5b-F	5′-GCAGCAGCTTATGCCAACTG-3′
qGmFT5b-R	5′-AGTATAAGCTAGAGTAGAGGCATCC-3
qGmFT4-F	5′-TCAACGTGAAGGTGGTTGGG-3′
qGmFT4-R	5′-CAGTGTGACTGCACCAAACG-3′
qGmFT6-F	5′-CCAGCCCAAGTGACCCAAAT-3′
qGmFT6-R	5′-GACAGCAGCAACTGGTAAGC-3′
qGmActin-F	5′-CGGTGGTTCTATCTTGGCATC-3′
qGmActin-R	5′-GTCTTTCGCTTCAATAACCCTA-3′
**For the semi-quantitative RT-PCR analysis**	
RT-PCR-GmFT1a-F	5'-CCTTTCACAAGTAGCGTTTCTATGG -3'
RT-PCR-GmFT1a-R	5'-ATATTCTCTCTGGGTTGGATTGCC -3'
RT-PCR-E1-F	5'-CACTCAAATTAAGCCCTTTCA-3'
RT-PCR-E1-R	5'-TTCATCTCCTCTTCATTTTTGTTG-3'
TUA5-F	5'-TGCCACCATCAAGACTAAGAGG -3'
TUA5-R	5'-ACCACCAGGAACAACAGAAGG -3'
**For transgenic plants checking**	
RT-GmFT1a-F	5'-GGAGCCTTTCACAAGTAGCGTTTCTA-3'
RT-GmFT1a-R	5'-AATCTCAGCAAAGTCTCTGGTGTT-3'
RT-Bar-F	5'-GCACCATCGTCAACCACTACATC-3'
RT-Bar-R	5'-CAGAAACCCACGTCATGCCAGTT-3'
**For *GmFT1a* genomic cloning**	
GmFT1as-1F	5'- AAGCTTAATTTCGTTCCTAAATA -3'
GmFT1as-1R	5'- ACAAAACTGATACCAACTAAACAT -3'
GmFT1am-1F	5'- TAAAACTCCCACGGTTACACAATA -3'
GmFT1am-1R	5'- GATCAGACATAATAACACGAAGGC -3'
GmFT1ah-1F	5'- TATTGGACGCCACTTTGCTTAAAT -3'
GmFT1ah-1R	5'- TCCTGACTTCTTTCACGTTTTTGT -3'
For DLA Assay	
GmFT1a pro- F(*Hind* III)	5′- AAGCTTAATTTCGTTCCTAAATA -3′
GmFT1a pro- R(*Bam* HI)	5′- GGATCCAATAAATTAATATCGATAAGTTGGT-3′
E1- F(*Sal* I)	5'- GTCGACATGAGCAACCCTTCAGATGAAAGGG-3'
E1- R(*Xba* I)	5'- TCTAGATTAATTCTCTGGCATAGCTTGTTT-3'
